# Extinction of influenza B Yamagata: Its impact on public health and vaccine implications

**DOI:** 10.7555/JBR.38.20240158

**Published:** 2024-08-22

**Authors:** Muhammad Awais Ashraf, Muhammad Asif Raza, Muhammad Nabeel Amjad

**Affiliations:** 1 CAS Key Laboratory of Molecular Virology & Immunology, Institutional Center for Shared Technologies and Facilities, Pathogen Discovery and Big Data Platform, Shanghai Institute of Immunity and Infection, Chinese Academy of Sciences, Shanghai 200031, China; 2 University of Chinese Academy of Sciences, Beijing, 101408, China

Dear Editor,

The circulation of respiratory viruses, primarily the influenza virus, has been disrupted by the emergence of the COVID-19 pandemic and SARS-CoV-2. Epidemiological surveillance data during the COVID-19 pandemic indicate a decline in the circulation of the influenza B Yamagata lineage in recent periods, suggesting a dynamic shift in the epidemiology of influenza B viruses (IBVs). Various factors may contribute to this decline, including changes in viral fitness, host immunity profiles, inter-lineage competition, and random fluctuations in transmission. However, as the virus undergoes repeated antigenic changes, its ability to effectively infect hosts and spread diminishes, leading to the decline and potential extinction of the Yamagata lineage strains. Competition with other influenza strains, such as alternate influenza B lineages and influenza A viruses, may also play a role in the extinction of the Yamagata lineage.

The Victoria lineage has emerged as a prominent circulating strain, exhibiting a higher prevalence and genetic diversity, compared with the Yamagata lineage in certain regions. These variations highlight the complex interplay of evolutionary forces, antigenic selection pressures, and ecological dynamics that shape influenza B virus epidemiology at local, regional, and global scales^[1]^. Before the emergence of SARS-CoV-2, the B/Victoria and B/Yamagata influenza virus lineages typically co-circulated, with no consistent pattern in their alternation from one influenza season to the next. Between 2012 and 2017, B/Yamagata viruses caused more infections globally than B/Victoria viruses^[2]^. However, in the two years preceding the COVID-19 pandemic, the B/Victoria lineage became more prevalent, leading to a shift in the ratio of B/Yamagata to B/Victoria dropping to 1∶4.5 in 2018 and 1∶19.3 in 2019^[2]^.

Vaccination strategies against IBV strains, specifically the Yamagata and Victoria lineages, are crucial for protecting human populations from seasonal flu outbreaks. These strategies primarily involve the development and distribution of quadrivalent influenza vaccines (QIVs), which contain antigens from both influenza B virus lineages as well as two influenza A strains^[3]^. By incorporating both Yamagata and Victoria strains into the vaccine, these strategies ensure a broader protection and an increased vaccine efficacy, because the prevalence of each lineage may vary from season to season. There are two main types of influenza vaccines available: inactivated influenza vaccines (IIVs) and live attenuated influenza vaccines (LAIVs). IIVs are administered *via* injection and are suitable for a wide range of individuals, including those with compromised immune systems. LAIVs, on the other hand, are administered as a nasal spray and are typically recommended for healthy individuals aged 2–49 years who are not pregnant^[4]^. Targeted vaccination campaigns focus on high-risk groups, such as the elderly, children, pregnant women, and individuals with chronic health conditions, who are more vulnerable to severe flu complications. Public health initiatives also promote annual vaccination for the general population to maintain high levels of immunity within the community, thereby reducing transmission rates.

During the early stages of the COVID-19 pandemic (starting in March 2020), and even when influenza viruses began circulating again in late 2021, B/Yamagata viruses were detected in only a few countries. This raised concerns that the B/Yamagata lineage might be nearing extinction^[5–7]^. However, declaring the extinction of the B/Yamagata lineage would be premature. The virus might be circulating at low levels, below the detection thresholds of current surveillance systems, or in regions not well covered by these systems, leaving room for a potential resurgence. For example, during much of the 1990s, the B/Victoria lineage was mostly absent from many regions worldwide and was mainly confined to East Asia before spreading globally again in the early 2000s^[8]^. The current situation has significant public health implications, particularly for influenza vaccination strategies. QIVs include strains from both the B/Yamagata and B/Victoria lineages. This raises the question of whether it is necessary to include a strain that is not currently circulating. Moreover, there is a concern that the mechanistically attenuated replication of the B/Yamagata strains in the LAIV, primarily administered to children, could lead to the reintroduction of the hemagglutinin and neuraminidase genes of a potentially extinct virus through reassortment with a co-infecting wild-type B/Victoria virus^[9]^. However, despite the LAIV retaining its cold-adapted nature and having a low potential for secondary infections from vaccine-derived influenza B/Yamagata infections, no such incidents have been reported to date^[10]^. Continuous monitoring of viral circulation remains crucial in adapting influenza prevention policies, particularly in optimizing vaccine formulation and composition, to align with the current epidemiological landscape.

We collected data from the World Health Organization (WHO) FluNet and the Global Initiative on Sharing All Influenza Data (GISAID) database, spanning from January 1, 2019, to April 30, 2024. Our analysis revealed a significant decline in influenza activity following the emergence of SARS-CoV-2. However, there was a significant increase in influenza activity from autumn 2023 onwards, indicating a resurgence in influenza circulation. Furthermore, ***Table 1*** presents an analysis of the B/Yamagata hemagglutinin genome sequences uploaded to the GISAID database from 2019 through April 2024. The table illustrates a consistent decline in the number of B/Yamagata sequences, both in terms of total uploads and the number of countries reporting them, starting in 2019. According to the annual reports for 2020–2021 and 2022–2023 of the Global Influenza Hospital Surveillance Network, all detected influenza cases were influenza A, with only rare cases of influenza B, all belonging to the B/Victoria lineage. No B/Yamagata cases were detected during these surveillance periods.

**Table 1:** Influenza B/Yamagata virus hemagglutinin segment sequences by the year of specimen collection

Year	IBV/Yamagata sequence (*n*)	Number of countries	Number of sequences by country
2019	988	74	Too many to list here
2020	119	14	United States (75), France (10), Tunisia (8), Norway (6), Russia (5), Chile (4), Trinidad and Tobago (3), Australia (2), Germany (1), Honduras (1), Jamaica (1), Japan (1), South Korea (1), Spain (1)
2021	0	0	NA
2022	0	0	NA
2023	0	0	NA
2024	0	0	NA
Data were obtained from the Global Initiative on Sharing All Influenza Data database between January 1, 2019 and April 30, 2024.Abbreviations: IBV, influenza B virus; NA, not available.			

Notably, no influenza B/Yamagata sequences have been uploaded with specimen collection dates after March 2020, indicating a cessation in the circulation of this lineage. The absence of B/Yamagata sequences post-March 2020 highlights the importance of continued molecular surveillance to monitor lineage dynamics and detect potential resurgence events. Overall, the combined analysis of epidemiological and molecular surveillance data provides valuable insights into the temporal trends and dynamics of influenza activity, informing strategies for influenza prevention, control, and preparedness^[11–13]^.

***Table 2*** highlights the percentage of influenza B virus cases worldwide from 2019 to 2023, which ranged between 16% and 20%. Notably, within this 20%, no influenza B/Yamagata cases were detected. A major decline in B/Yamagata cases was observed after 2021. In 2021, 41 cases were reported, with most originating from China, but none of the sequences were uploaded to GISAID. According to the WHO FluNet database, the most recent case was reported in Cuba in 2023.

**Table 2:** Total influenza cases including influenza A and B from 2019 to 2023

Year	Total influenza (*n*)	IAV (*n*)	%	IBV (*n*)	%	IBV/Victoria (%)	IBV/Yamagata (%)
2019	789011	657369	83.3	131642	16.7	92.9	7.1
2020	471106	303038	64.3	168068	35.7	98.6	1.4
2021	116225	82181	70.7	30044	29.3	99.9	0.1
2022	363251	329688	90.8	33563	9.2	100	0
2023	892260	712497	79.9	179763	20.1	100	0
Abbreviations: IAV, influenza A virus; IBV, influenza B virus.							

Seasonal influenza outbreaks are primarily driven by the influenza A and B virus types. A comprehensive study spanning 29 nations revealed that approximately 23% of influenza cases were attributed to the influenza B virus^[14–15]^. Notably, influenza B emerged as the dominant virus type in roughly one out of every seven seasonal outbreaks. Two genetically distinct influenza B lineages, namely B/Victoria/2/87 (B/Victoria) and B/Yamagata/16/88 (B/Yamagata), first emerged in the 1970s and have co-circulated globally, with both lineages remaining active at least since 2001^[1]^. Before 2012, influenza vaccines primarily followed a trivalent composition, containing one strain of influenza A(H3), one strain of influenza A(H1) or A(H1) pdm09 (post-2010), and one strain of influenza B. The unpredictable circulation of influenza B/Victoria and B/Yamagata lineages posed challenges in vaccine strain selection, leading to frequent mismatches. These mismatches occurred in over 40% of seasons in temperate regions and 30% in tropical areas^[16]^. Additionally, the antigenic differences between the two lineages reduced cross-protection. To tackle this issue, QIVs were introduced in February 2012, incorporating both B/Yamagata and B/Victoria lineages. QIVs were subsequently approved by the European Medicines Agency in 2013. However, the potential disappearance of B/Yamagata may significantly impact the continued use of QIVs, which have become prevalent in Europe. Future scenarios may include maintaining QIVs with a novel strain or reverting to trivalent influenza vaccines. FluNet database analysis revealed a significant decline in influenza B/Yamagata cases in 2021 and 2022, though sporadic detections continued in various regions. These isolated occurrences raise concern, as they may originate from vaccine-derived sources, potentially linked to LAIV or RNA remnants from inactivated vaccines administered near the time of specimen collection. It is crucial to interpret B/Yamagata detections with caution, as they may not necessarily indicate the circulation of wild-type B/Yamagata virus without thorough virological and epidemiological analysis. The failure to sequence and characterize these B/Yamagata detections impedes efforts to accurately determine whether the lineage has gone extinct. The lack of B/Yamagata sequencing data underscores a necessity for the enhanced laboratory capabilities worldwide. Implementing in-house-developed lineage-specific multiplex RT-PCR assays could aid primary diagnostic laboratories in determining the lineage of detected influenza B viruses. Furthermore, collaboration between primary diagnostic laboratories and National Influenza Centers is crucial for comprehensive investigation and surveillance of influenza B viruses, particularly in monitoring the potential extinction of B/Yamagata lineage viruses^[17–18]^.

The analysis of the FluNet database and other sources indicates a significant decline in the reported B/Yamagata cases, although some data may be overestimated because of the dynamic nature of reporting systems. The under-representation of data from tropical regions, where influenza B previously circulated more than in temperate regions, is a key limitation. As the disappearance of B/Yamagata could shift the age distribution of influenza B cases and potentially reduce the disease burden, continuous epidemiological and virological monitoring is crucial. Furthermore, the potential extinction of the B/Yamagata lineage presents unprecedented challenges for international, regional, and national public health authorities. The current study aims to support decision-making processes regarding vaccine composition and public health strategies, emphasizing the need for sustained vigilance in monitoring respiratory viruses to protect global health.

We extend our deepest gratitude to FluNet and GISAID for generously granting us the access to their invaluable databases. We also sincerely appreciate all the dedicated contributors who have shared their data and sequences with these esteemed repositories.

Yours Sincerely,Muhammad Awais Ashraf^1,2,✉^, Muhammad Asif Raza^1,2^, Muhammad Nabeel Amjad^1,2^ ^1^CAS Key Laboratory of Molecular Virology & Immunology, Institutional Center for Shared Technologies and Facilities, Pathogen Discovery and Big Data Platform, Shanghai Institute of Immunity and Infection, Chinese Academy of Sciences, Shanghai 200031,China;^2^University of Chinese Academy of Sciences, Beijing 101408,China.^✉^Corresponding author: Muhammad Awais Ashraf. E-mail: awais@siii.cas.cn.
